# Hip Involvement in Juvenile Idiopathic Arthritis: A Roadmap From Arthritis to Total Hip Arthroplasty or How Can We Prevent Hip Damage?

**DOI:** 10.3389/fped.2021.747779

**Published:** 2021-11-05

**Authors:** Lubov S. Sorokina, Ilia S. Avrusin, Rinat K. Raupov, Natalia A. Lubimova, Sergey V. Khrypov, Mikhail M. Kostik

**Affiliations:** ^1^Department of Hospital Pediatry, Saint-Petersburg State Pediatric Medical University, Saint Petersburg, Russia; ^2^Leningrad Regional Children's Hospital, Saint Petersburg, Russia; ^3^Saint Petersburg State Health Care Establishment the City Hospital, Saint Petersburg, Russia; ^4^Almazov National Research Medical Centre, Saint Petersburg, Russia; ^5^St. Petersburg Clinical Scientific and Practical Center of Specialized Types of Medical Aid (Oncological), Saint Petersburg, Russia

**Keywords:** juvenile idiopathic arthritis, hip osteoarthritis, total hip arthroplasty, corticosteroids, avascular osteonecrosis of the femoral head

## Abstract

**Objectives:** To describe the clinical characteristics of hip involvement in juvenile idiopathic arthritis (JIA) from arthritis to hip osteoarthritis (HOA) and total hip arthroplasty (THA).

**Study Design:** Seven hundred fifty-three patients aged 2–17 years with JIA were included in the study. The comparison analysis was performed between the following subgroups: (i) JIA without hip involvement (*n* = 600; 79.7%) vs. JIA with hip involvement without HOA (*n* = 105; 13.9%), (ii) JIA with hip involvement with HOA, but without THA (*n* = 32; 4.3%) and JIA with hip involvement with HOA and with THA (*n* = 16; 2.1%). Clinical, laboratory characteristics and treatment regimens compared.

**Results:** Hip involvement was present in 20.3% of patients. HOA was present in 6.4% (12^*^1,000 patient-years) of the entire JIA group and 31.4% of patients with hip involvement. Sixteen patients (2.1%; 4.0^*^1,000 patient-years) required THA. The following factors were associated with HOA: sJIA (OR = 3.6, *p* = 0.008; HR = 3.0, *p* = 0.002), delayed remission (OR = 4.2, *p* = 0.004; HR = 1.4, *p* = 0.538), delay in biologic therapy initiation (OR = 7.5, *p* = 0.00001; HR = 6.7, *p* = 0.002), alkaline phosphatase <165 U\l (OR = 4.1, *p* = 0.0003; HR = 5.2, *p* = 0.000004), treatment with corticosteroids (CS) (OR = 2.6, *p* = 0.008; HR = 1.2, *p* = 0.670), cumulative corticosteroids >2,700 mg (OR = 4.3, *p* = 0.032; HR = 1.4, *p* = 0.527). The following factors were associated with THA: delay in biologic treatment initiation (OR = 1.04, *p* = 0.0001; HR = 9.1, *p* = 0.034), delayed hip involvement (OR = 5.2, *p* = 0.002; HR = 3.0, *p* = 0.044), and methylprednisolone pulse therapy (OR = 10.8, *p* = 0.0000001; HR = 5.6, *p* = 0.002).

**Conclusion:** Both sJIA and systemic CS, impaired calcium-phosphorus metabolism, and delayed hip arthritis are associated with HOA development in JIA. HOA is considered to be a severe adverse event of CS treatment, especially delayed hip involvement.

## Introduction

Juvenile idiopathic arthritis (JIA) encompasses seven different subtypes according to International League Against Rheumatism (ILAR) classification, but in real clinical practice, it is often divided into systemic and non-systemic groups, which differ in clinical features and treatment approach (1, 2). Many delayed complications, i.e., linear growth retardation, osteoporosis, and severe functional disability due to secondary structural changes in the adjacent bone and osteoarthritis formation (3, 4), are associated with the chronic course of the disease and inadequate therapy. Hip joint is one of the most frequently damaged joints in JIA, manifested in pain, severe walk disturbances, and total hip arthroplasty (THA) is required even in childhood in case of end-stage hip osteoarthritis (HOA) (5). There is little data on specific predictors of HOA development in different JIA subtypes. The introduction of steroid-sparing agents, such as methotrexate and biologics, into treatment regimens, dramatically influenced the course and outcomes of the disease. However, structural changes of joints, primarily in the hip, do still occur despite our current therapies (6). Nowadays, most JIA patients undergoing THA have shifted to an older age group (7, 8). Known HOA risk factors are not specific: early onset of the disease, early radiological hip changes, and higher baseline activity and inflammation. Chronic immune-related joint inflammation and corticosteroid usage are tightly connected with the risk of avascular necrosis (AVN) (9). Corticosteroids are considered the leading risk factor of AVN in adults, but there are few data in children. The association of AVN development with corticosteroid dosing was described well in juvenile systemic lupus erythematosus (SLE) and oncohematological disorders (10, 11). Anecdotally, hip osteoarthritis due to AVN is studied better in SLE than in JIA, although JIA is the more common. It is worth noting that both AVN and end-stage HOA are treated by total joint replacement, which is still a challenge in pediatric practice. Our study aimed to describe the clinical characteristics of hip involvement in juvenile idiopathic arthritis (JIA) from arthritis to hip osteoarthritis (HOA) and total hip arthroplasty (THA).

## Methods

### Ethical Expertise

Written consent was obtained according to the declaration of Helsinki. The local Ethical Committee of Saint Petersburg State Pediatric Medical University (protocol number 11/10 from 23.11.2020) approved this retrospective study's protocol.

### Study Design and Patient Selection

Seven hundred fifty-three patients with JIA aged 2–17 years were included in a retrospective single-center study between January 2007 and December 2016. Diagnosis of JIA and JIA subtypes was made according to ILAR criteria (1). Inclusion criteria: (i) all subtypes of JIA according to ILAR criteria; (ii) at least two evaluations in our center; (iii) data about disease onset and course if the patient had an initial observation in another center; (iv) data about at least 2 year-course was available. Exclusion criteria: (i) the presence of overlap immune-mediated diseases (inflammatory bowel disease, chronic non-bacterial osteomyelitis), (ii) a history of non-inflammatory hip pathology (congenital dislocation of the hip, dysplastic osteoarthritis, acetabular dysplasia, post-traumatic changes).

**Figure 1:**
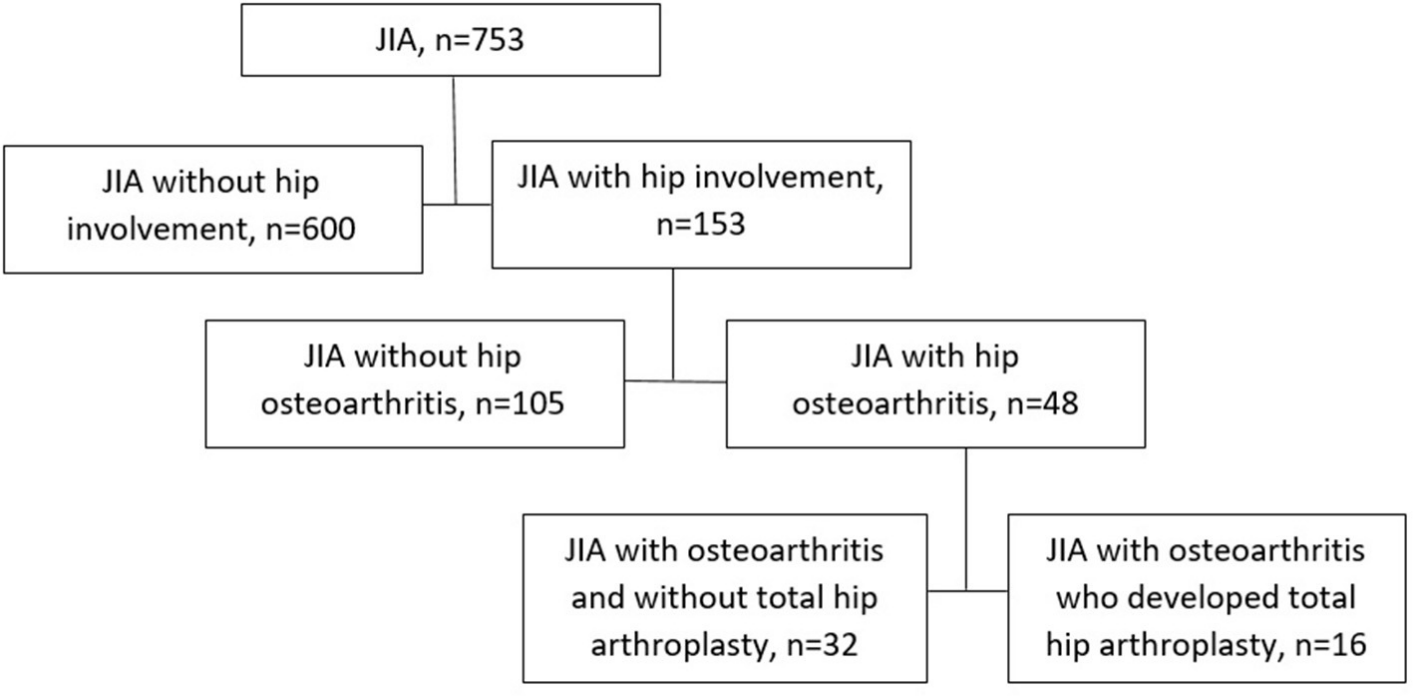
The distribution of the enrolled patients, according to the stage of hip involvement.

Patients were divided into four categories (Figure 1), according to the stage of hip involvement: (i) patients who underwent THA due to HOA (*n* = 16), (ii) patients with HOA without indications for THA (*n* = 32), (iii) patients with hip arthritis (HA) without femoral head structural changes (*n* = 105), and (iv) patients without hip arthritis (*n* = 600). Diagnosis of HOA was established according to Dale radiographic JIA classification for interpretation of hip radiograms: grade 0 (normal joints), grade 1 (juxta-articular osteoporosis and/or periarticular soft tissue swelling), grade 2 (growth disturbance), grade 3 (growth abnormality and marginal bony erosions), grade 4 (deformation and severe erosions), and grade 5 (gross destruction and deformation) (12). Hip arthritis (HA) was diagnosed based on synovial inflammation with or without joint effusion on magnetic resonance imaging (MRI) or/and ultrasound examination *without specific radiological changes on X-ray (Dale classification grades 0–2)*. Hip osteoarthritis was diagnosed beginning with grade 3 by Dale classification if such signs as erosions, deformation, flattening of the femoral head, joint space narrowing, and sclerosis in the joint-forming bones were detected by radiological examination. MRI or computer tomography (CT) examination was performed for each patient with HOA. Every patient with hip involvement had at least two radiological examinations. All x-rays, CT scans, and MRIs were reassessed by an experienced pediatric rheumatologist (MK) and orthopedic surgeon (SK) with 30+ years of experience in THA. All doubtful cases, were excluded from the study (*n* = 354). Data of the WOMAC scale, Oxford hip score, hip pain severity, inability to walk, and patients' and parental consent were the indications for THA. In patients with THA, a histological examination was performed.

### Data Collection: We Evaluated

*Demographic characteristics*: gender, onset age, JIA category, type of hip involvement, the time before HOA and THA, number of patients with delayed hip involvement. Delayed hip involvement means the absence of hip involvement in the first 6 months since the JIA onset.

*Laboratory activity* at the last visit (hemoglobin, WBC, platelets, ESR, and CRP levels) were included in the analysis.

*Bone metabolism* (calcium, inorganic phosphate, alkaline phosphatase, parathyroid hormone, and 25OHD levels) in the same time points as a laboratory activity.

*Treatment regimens*, cumulative corticosteroid doses, the route of administration, biologic and non-biologic DMARDs.

*Achievement* of inactive disease and significant flare (lead to change treatment).

### Statistical Analysis

Sample size was not calculated initially. Statistical analysis was performed with the software STATISTICA, version 10.0 (StatSoft Inc., USA). All continuous variables were checked by the Kolmogorov-Smirnov test, with no normal distribution identified. The quantitative variables were median (Me) and percentiles (25; 75) for continuous variables and absolute frequencies and percentages for categorical variables. Pearson's χ2 test or the Fisher's exact test in the expected frequencies <5 was used to compare the categorical variables. Two quantitative variables were compared using the Mann-Whitney test and Kruskal-Wallis Anova test if the number of variables was more than two. The ability of each variable to discriminate HOA from hip arthritis without structural changes was evaluated with sensitivity and specificity analysis, AUC-ROC (area under the receiver operating characteristic curve) with 95% confidence interval (CI), calculating odds ratio (OR) for the detection the best cut-offs of continuous variables. The higher values of OR of variables interfere with the better discriminatory ability. We used the “best” threshold for our data's ROC curve analysis because it provides the most appropriate mean between sensitivity and specificity. Survival analysis in each group, with HOA as the event of interest, was conducted through the Kaplan-Meier method. The log-rank test compared survival curves. Factors significantly associated with the time of HOAonset or achievement of the remission was then tested in a Cox proportional hazards regression model, calculating the Hazard-ratio (HR) with a 95% confidence interval (95%CI). *P* < 0.05 was considered statistically significant.

## Results

**Figure 2:**
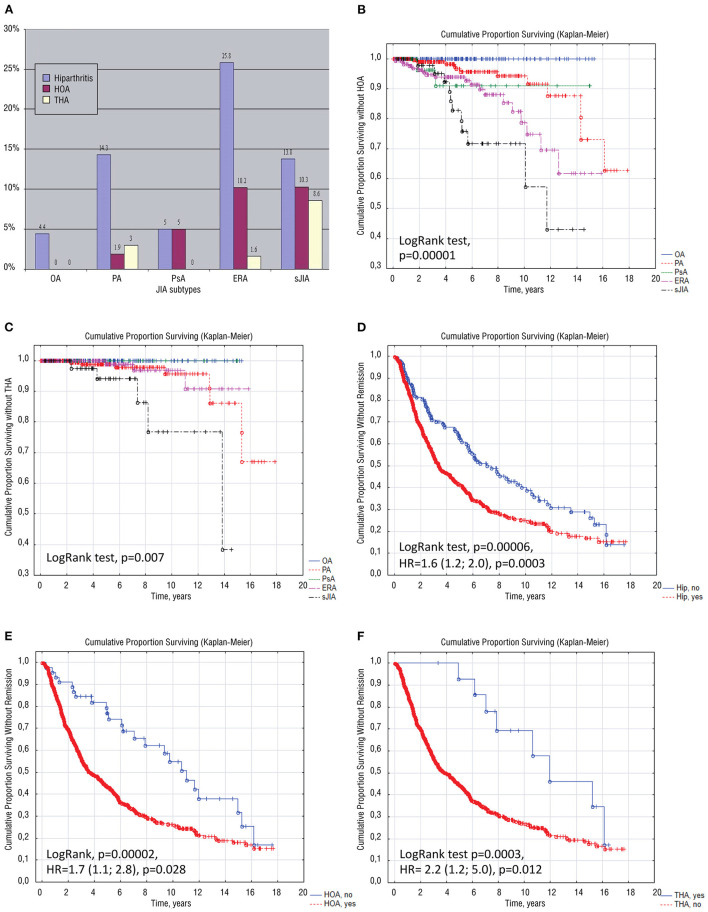
Multiple schemes are depicting the distribution of different types of hip involvement in children with JIA categories **(A)**, the cumulative probability of surviving without hip osteoarthritis **(B)** and without total hip arthroplasty **(C)** in children with distinct JIA categories; Cumulative probability of achievement of remission in JIA depending on the hip involvement **(D)**, on the development of hip osteoarthritis **(E)** and depending on total hip arthroplasty **(F)**. ERA, enthesitis-related arthritis; HOA, hip osteoarthritis; HR, hazard ratio; JIA, juvenile idiopathic arthritis; OA, oligoarthritis; PA, polyarthritis; PsA, psoriatic arthritis; sJIA, systemic JIA; THA, total hip arthroplasty.

Any type of hip involvement (hip arthritis, hip osteoarthritis, and total hip arthroplasty due to hip osteoarthritis) was found in 153 (20.3%) patients with JIA. Hip arthritis was detected more frequently in ERA (37.6%), systemic arthritis (32.6%), polyarthritis (19.2%), and rarely in psoriatic arthritis (10%), and oligoarthritis (4.2%). Hip osteoarthritis (with and without THA) developed in 48 (6.4%) of JIA patients, and it amounted to 31.4% of patients with hip involvement. Bilateral hip involvement was in 22 (45.8%) of HOA patients, in total 70/96 (72.9%) of hips were involvement. HOA frequently occurred in patients with systemic arthritis and ERA compared to polyarthritis and psoriatic arthritis. No cases of hip osteoarthritis in oligoarthritis were observed. The third of the patients (51/153; 33%) had no hip arthritis at onset and developed it later. These patients had more frequent corticosteroid administration (39.2%) than patients with hip arthritis at the JIA onset (21.2%), *p* = 0.023. The time between JIA onset and HOA development ranged from systemic arthritis (4.5 years) to ERA (5.5 years) and polyarthritis (6.5 years) patients. Sixteen patients (2.1%, 4.0^*^1000PY) required THA: systemic arthritis (8.6%), polyarthritis (3%), and ERA (1.6%) through a similar time in ERA and sJIA (7 years) and through 11 years in polyarthritis. The time between HOA and THA was similar in all JIA categories, the shortest being in ERA (0.8 years) and the longest in sJIA (2.1 years). The distribution of different types of hip involvement is presented in Table 1 and Figure 2A. Significant differences in the cumulative probability of HOA development (Figure 2B) and THA (Figure 2C) were observed in different JIA categories. The probability of the development of HOA ranged from psoriatic arthritis (minimal) to polyarthritis, ERA, and sJIA (maximum), but the probability of THA ranged from ERA (minimum) to polyarthritis and sJIA (maximum).

**Table 1:** Hip involvement in children with JIA categories.

**Features of hip involvement, *n* (%)**	**Total** **(*n* = 753)**	**OA** **(*n* = 204)**	**PA** **(*n* = 265)**	**PsA** **(*n* = 40)**	**ERA** **(*n* = 186)**	**sJIA** **(*n* = 58)**	***p***
Female, *n* (%)	458 (60.8)	136 (66.7)	195 (73.6)	18 (45.0)	71 (38.2)	37 (63.8)	0.0000001
The age of inclusion in the study, years, Me [25–75%]	12.3 (7.9–16.5)	10.4 (6.3–14.3)	11.4 (7.1–16.2)	15.8 (10.6–17.6)	15.2 (12.1–17.6)	10.4 (6.7–14.7)	0.00001
Intact hip, *n* (%)	600 (79.7)	195 (95.6)	214 (80.7)	36 (90.0)	116 (62.4)	39 (67.2)	0.0000001
Any hip involvement, *n* (%)	153 (20.3)	9 (4.4)	51 (19.2)	4 (10.0)	70 (37.6)	19 (32.8)	0.0000001
Hip arthritis, *n* (%)	105/153^*^ (68.6)	9 (4.4)	38 (14.3)	2 (5.0)	48 (25.8)	8 (13.8)	0.0000001
All HOA, *n* (%)	48/153^*^(31.4)	0 (0.0)	13 (4.9)	2 (5.0)	22 (11.8)	11 (19.0)	0.0000001
Frequency of HOA,^*^1000 PY	12	0	8.6	10.6	20.3	37.1	0.0000001
HOA w/o THA, *n* (%)	32/153^*^ (20.9)	0 (0.0)	5 (1.9)	2 (5.0)	19 (10.2)	6 (10.3)	0.0000001
HOA with THA, *n* (%)	16/153^*^ (10.4)	0 (0.0)	8 (3)	0 (0.0)	3 (1.6)	5 (8.6)	0.0000001
HOA with THA, ^*^1000PY	4.0	0	5.3	0	2.8	16.9	0.0000001
THA of HOA (%)	33.3	na	61.5	na	13.6	45.5	0.0000001
Time to HOA, years, Me [25–75%]	5.0 (2.4–9.4)	na	6.5 (4.4–13)	2.5 (1.8–3.2)	5.5 (1.5–9.0)	4,5 (3.9–5.7)	0.649
The time between JIA onset and THA, years, Me [25–75%]	7.8 (4.6–12.9)	na	11.2 (4.4–14.1)	na	7.1 (5.0–11.0)	7.4 (4.3–8.2)	0.905
The time between HOA and THA, years, Me[25–75%]	1.4 (0.6–2.3)	na	1.4 (1.0–3.2)	na	0.8 (0.5–1.8)	2.1 (0.4–2.5)	0.970

* *Calculated to patients who had hip involvement (n = 153), CS, corticosteroids; ERA, enthesitis-related arthritis; HOA, hip osteoarthritis; JIA, juvenile idiopathic arthritis; na, not applicable; OA, oligoarthritis; PA, polyarthritis; PsA, psoriatic arthritis; PY, patient-years; sJIA, systemic JIA; THA, total hip arthroplasty*.

Patients with any hip involvement had later JIA onset age, longer JIA course, higher laboratory inflammatory activity (CRP and ESR levels, anemia, leuko-, and thrombocytosis), a higher number of clinically active joints, high distribution of HLA B27, and more often belonged to the ERA JIA category. The highest proportion of the patients with bilateral hip involvement was observed in patients whom THA was undergone and the lowest in the patients with hip arthritis (Table 2). Patients with hip involvement had cervical spine involvement and arthritis of the upper body, higher frequency of all types of corticosteroids usage: systemic oral, pulse therapy and hip intra-articular as well as higher corticosteroid cumulative doses, high frequency of biologics, more extended period before achievement of remission and lower probability of remission [Log Rank test, *p* = 0.00006, HR = 1.6 (95%CI: 1.2; 2.0), *p* = 0.0003, Table 2; Figure 2D]. No significant differences in total and ionized calcium and 25OHD levels between patients with different types of hip involvement were observed, but in logistic regression analysis, Ca <2.42 mmol/l was associated with HOA: OR = 4.2 (95%CI: 1.7; 10.2), *p* = 0.0006, RR = 3.1 (95%CI: 1.3; 7.4), *p* = 0.012. Alkaline phosphatase levels were lower in THA 165 (121; 412) U/l and HOA groups 123 (79; 182) U/l, compared to patients with hip arthritis 207 (143; 352) U/l and with no hip involvement 225 (147; 390) U/l (*p* = 0.001). Subsequent analysis was done with four different types of hip involvement (Table 2).

**Table 2:** The features of juvenile idiopathic arthritis in different types of hip involvement.

**JIA features**	**THA (*n* = 16)**	**HOA (*n* = 32)**	**Hip arthritis (*n* = 105)**	**All hip involvement** **(*n* = 153)**	**No hip arthritis (*n* = 600)**	**Total, *n* = 753**	**p_**1**_**	**p_**2**_**
Females, *n* (%)	9 (56.3)	19 (59.4)	50 (47.6)	78 (51)	379 (63.2)	457 (60.7)	0.006	0.266
Europeans, *n* (%)	14 (87.5)	26 (81.3)	88 (83.8)	128 (83.7)	536 (89.5)	664 (88.3)	0.045	0.221
Onset age, years, Me [25–75%]	8.0 (3.5–11)	8.3 (4.3–13)	7.4 (4.0–11.5)	7.6 (4.0–11.6)	5.5 (2.8–10.1)	6.0 (3.0–10.4)	0.00017	0.0004
JIA duration, years, Me [25–75%]	8.5 (6.5–3.2)	5.4 (2.8–11.0)	6.2 (3.7–9.5)	6.4 (3.4–10.3)	3.7 (1.5–6.8)	4.3 (1.9–7.5)	0.0000001	0.0001
**JIA subtypes** Oligoarthritis, *n* (%) Polyarthritis, *n* (%) Psoriatic, *n* (%) ERA, *n* (%) sJIA, *n* (%)	0 (0) 8 (50) 0 (0) 3 (18.7) 5 (31.2)	0 (0) 5 (15.6) 2 (6.2) 19 (59.4) 6 (18.7)	9 (4.4) 38 (14.3) 2 (5) 48 (25.8) 8 (13.8)	9 (5.9) 51 (33.3) 4 (2.6) 70 (45.7) 19 (12.4)	195 (95.6) 214 (80.7) 36 (90) 116 (62.4) 39 (67.2)	204 (27.1) 265 (35.2) 40 (5.3) 186 (24.7) 58 (7.7)	0.0000001	0.0000001
**Laboratory** ANA, *n* (%) HLAB27, *n* (%)	3/8 (37.5) 3/6 (50)	5/16 (31.3) 9/19 (47.4)	23/59 (39.0) 22/50 (44.0)	31/83 (37.3) 34/75 (45.3)	181/377 (48) 66/233 (28.3)	212/460 (46.1) 100/308 (32.5)	0.008 0.004	0.173 0.0007
**Active joints, n** Hip arthritis at onset, *n* (%)	22 (9; 53) 5 (31.3)	9 (5; 16) 12/29 (41.4)	9 (5; 18) 52/90 (57.8)	10 (5; 20) 69 (45.1)	5 (3; 10) na	6 (3; 12) 69/153 (45.1)	0.0000001 na	0.00001 0.07^*^
Bilateral involvement, *n* (%) Number involved hips, *n* (%)	13 (81.3) 29/32 (90.6)	18 (56.3) 50/64 (78.1)	44 (44.5) 149/210 (71.0)	75 (49.0) 228/306 (74.5)	na na	75 (10.0) 228/1506 (15.1)	na na	0.009^*^ 0.013^*^
**Treatment** Intra-articular CS, *n* (%) Hip CS injection, *n* (%) Oral CS, *n* (%) Pulse-therapy CS, *n* (%) Any CS, *n* (%) Cumulative CS, grams, Me [25–75%] Methotrexate, *n* (%) Biologic, *n* (%) Time to biologic, years Me [25%;75%]	6 (37.5) 0 (0) 9 (56.2) 11 (68.8) 15 (93.8) 5.0 (3.0–14.0) 14 (87.5) 15 (93.8) 7.5 (4.4–11.4)	5 (15.6) 2 (6.3) 12 (37.5) 10 (31.2) 16 (50) 4.5 (0.5–2.0) 26 (81.3) 28 (87.5) 4.0 (1.9–8.6)	27 (25.7) 5 (4.8) 24 (22.9) 25 (23.8) 53 (50.5) 3.0 (1.3–6.0) 77/95 (73.3) 56 (53.3) 4.9 (2.2–7.6)	38 (24.8) 7 (4.6) 45 (29.4) 46 (30.1) 84 (54.9) 3.5 (1.5–7.8) 117 (76.5) 99 (64.7) 4.8 (2.3–8.4)	276 (46) na 107 (17.9) 89 (14.8) 361 (60.2) 1.5 (1.0–3.8) 456 (76.0) 252 (42.0) 4.1 (1.7–7.7)	314 (41.7) 7/153 (4.6) 152 (20.2) 135 (17.9) 445 (59.1) 2.7 (1.0–5.0) 573 (76.1) 351 (46.6) 4.2 (1.9–7.8)	0.000002 na 0.002 0.00001 0.37 0.003 0.372 0.000001 0.09	0.00002 0.0000001^*^ 0.00009 0.0000001 0.006 0.0014 0.438 0.0000001 0.128
**Outcomes** Remission, *n* (%) Time to remission, years Me [25%;75%] Flare, *n* (%)	8 (50.0) 9.3 (6.6–15.4) 0 (0)	16 (50.0) 5.6 (3.3–11.4) 0 (0)	63 (60.0) 4.8 (1.5–8.0) 13 (12.4)	87 (56.9) 5.6 (2.4–9.5) 13 (8.5)	398 (66.3) 2.9 (1.4–5.9) 124 (20.7)	485 (64.4) 3.2 (1.5–6.6) 137 (18.2)	0,029 0.000001 0.002	0.102 0.00001 0.016

*p_1_, comparison of all patients with all types of hip involvement (n = 153) with no hip involvement (n = 600); p_2_, comparison between four groups: THA (n = 16), HOA (n = 32); HA (n = 105) and no HA (n = 600)*;

* *p-value calculated for three groups: THA, HOA, HA. CS, corticosteroids; ERA, enthesitis-related arthritis; HA, hip arthritis; HOA, hip osteoarthritis; JIA, juvenile idiopathic arthritis; Me, median; THA, total hip arthroplasty*.

HOA was associated with more frequent corticosteroids usage (oral and pulse-therapy), delayed hip involvement, lower levels of alkaline phosphatase, and decreased probability of remission, compared to patients with hip arthritis without structural changes [LogRank test *p* = 0.00002; HR = 1.7 (95%CI: 1.1; 2.8) *p* = 0.028; Figure 2E]. Patients with HOA frequently required biologic administration due to the severity of the JIA (Table 3).

**Table 3:** Factors, associated with hip osteoarthritis and total hip arthroplasty.

**Factors, associated with HOA**	**OR (95% CI)**	***p***	**HR (95% CI)**	***p***
Systemic JIA, yes	3.6 (1.4; 9.8)	0.008	3.0 (1.5; 6.0)	0.002
Delayed remission (>5 years), yes	4.2 (1.5; 11.6)	0.004	1.4 (0.5; 3.8)	0.538
Delay in biologic treatment initiation, yes	7.5 (2.8; 20.5)	0.00001	6.7 (2.0; 25.0)	0.002
Alkaline phosphatase <165 U/l, yes	4.1 (1.9; 8.8)	0.0003	5.2 (2.6; 11.1)	0.000004
Oral corticosteroids, yes	2.6 (1.3; 5.5)	0.008	1.2 (0.6; 2.1)	0.670
Methylprednisolone pulse-therapy, yes	2.5 (1.2; 5.1)	0.013	1.5 (0.8; 2.7)	0.239
Delayed hip involvement, yes	4.6 (2.2; 9.6)	0.00003	2.4 (1.3; 4.4)	0.005
Cumulative corticosteroids >2,700 mg	4.3 (1.1; 17.1)	0.032	1.4 (0.5; 4.3)	0.527
**Factors, associated with THA**				
Delay in biologic treatment initiation	1.04 (2.4; 136.2)	0.0001	9.1 (1.2; 71.4)	0,034
Methylprednisolone pulse-therapy	10.8 (3.7; 31.7)	0.0000001	5.6 (1.9; 16.7)	0.002
Delayed hip involvement	5.2 (1.7; 15.8)	0.002	3.0 (1.03; s9.1)	0.044

*CI, confidence interval; JIA, juvenile idiopathic arthritis; HR, hazard ratio; HOA, hip osteoarthritis; OR, odds ratio; THA, total hip arthroplasty*.

Bilateral THA was undergone in 4 (25%) and unilateral in 12 (75%) of patients, in total 20 (62.5%) of hips were replaced. Patients who underwent THA also had increased inflammation, more active joints, treatment with methylprednisolone pulse-therapy, decreased probability of achievement of remission, and delay in biologic therapy initiation [Log Rank test *p* = 0.0003, HR = 2.2 (95%CI: 1.2; 5.0), *p* = 0.012, Table 2; Figure 2F]. Factors associated with THA were: delay in biologic therapy initiation, delayed hip involvement, and methylprednisolone pulse therapy (Table 3).

## Discussion

JIA is a chronic immune-mediated joint disease of childhood. There are no specific predictors for the long-term outcome for every subtype, only the general ones: early onset age of the disease, initial high activity, and female gender, which are considered unfavorable prognostic markers (13, 14). A research group from Canada created an alternative view of ILAR classification that highlighted hip involvement as a predictor of poor outcomes (15).

Our results support these findings. Patients with hip involvement had lower chances for remission and a longer time before it, their disease activity was higher, requiring corticosteroid therapy and more biologics compared to others. Hip involvement often occurred in systemic, enthesitis-related and polyarticular JIA categories with the highest rate of HOA and THA development. Shorter time of HOA development, delayed hip involvement, need of corticosteroids, and uncontrolled inflammation were hallmarks of HOA in sJIA, whereas male gender, older age, HLA B27-positivity, spine involvement, hip arthritis in the onset together with the shortest time between HOA and THA were markers of HOA in ERA. The main predictors of HOA and THA in JIA children were uncontrolled inflammation, higher total dose of corticosteroids and delayed initiation of biologic treatment. So, we have a chance to prevent HOA and THA by correction of the above mentioned HOA and THA predictors.

In the literature, hip arthritis occurred in 20–50% of patients with non-systemic JIA and 20–73% of patients with sJIA (15), and we noted the same rate in our study: 19.3 and 32.8%, respectively. If sJIA occurs before 10 years, it has a higher hip damage rate (15.1%) than in patients who develop sJIA after 10 years (7.1%) (3, 16). If sJIA started before 6 years, it had more frequent radiographic changes than the older group (17). In our cohort, opposite data was observed: patients older than 7.5 years had a higher probability of HOA [HR = 6.0 (95%CI: 2.7; 13.5), *p* = 0.00001 for the whole cohort, and OR = 5.8 (95%CI: 1.4; 24.0), *p* = 0.009 for sJIA]. The long study period included patients from the “pre-biologic era” (before the 2010 year), which might influence our results, e.g., higher onset age, compared to previous studies. Hip involvement is often bilateral and delayed in the literature-on average, hip arthritis develops 2 years after the disease starts, and HOA develops 6 years later with typical radiological changes (18, 19). The time gap between JIA onset and HOA development in our group was similar (an average of 5 years) but depended on the JIA category. The known risk factors of HOA in sJIA are early onset age of the disease, persistent inflammation, generalized lymphadenopathy, polyarthritis, and thrombocytosis over 600 × 10^9^/l (20, 21). In our study, the main predictors of HOA were systemic corticosteroids, systemic arthritis, delayed remission, and delay in biologic treatment initiation, together with decreased bone metabolism and delayed hip involvement is mentioned as having an ischemic pathway of hip damage. In sJIA subgroup the main predictors in the multiple regression analysis were onset age (*p* = 0.049) and corticosteroids >2,700 mg (*p* = 0.008) and white blood cell number (*p* = 0.024) and in non-systemic forms of JIA–corticosteroids >2,700 mg (*p* = 0.012), alkaline phosphates (*p* = 0.013) and calcium level (*p* = 0.026). Delayed hip involvement was associated with high disease activity and poor prognosis. Such patients received more steroids in the “pre-biologic era,” which was one of the predictors of subsequent femoral head avascular necrosis. Differences in bone metabolism were associated predominantly with systemic corticosteroids, especially decreased alkaline phosphatase activity. No differences in 25OHD might be explained by a more thorough control of vitamin D supplementation for children treated with systemic steroids, glucocorticoid-induced osteoporosis, and HOA formation.

Hip avascular necrosis (AVN) can be either idiopathic or a complication of the various illnesses (22). It is caused by a depressed blood supply in the bones due to various reasons. Predominantly it occurs in overloaded parts of the skeleton: femoral head, knee, and tarsus (23). Mostly, it is a problem of adult patients but occurs during childhood too. The pathway of corticosteroid-induced AVN is not fully understood. Vasculopathy, hypercoagulation, and overweight are discussed (24). The most sensitive childhood period is the time of growth plate closure because of significantly reducing blood supply and risk of ischemia. Our study noted that patients with sJIA developed HOA earlier than patients with other JIA subtypes, and it was associated with an aggressive course and prolonged corticosteroid treatment, with earlier disease onset and higher inflammatory activity. Patients with polyarticular subtype had the highest corticosteroid use and THA level within nsJIA. Patients with sJIA received more systemic steroids than other JIA categories, in which intra-articular steroids were more frequent. Different pathological mechanisms in systemic and non-systemic patients lead to HOA: side effects of systemic corticosteroids in sJIA lead to AVN and inflammation (osteitis) in ERA patients. Fast progression to HOA in sJIA leads to THA in childhood, and ERA progression is usually slower. Systemic arthritis, corticosteroids, delayed remission, related to delayed biologic administration in “pre-biologic era.” Control of inflammation, steroid avoidance, and “on time” biologic administration highlights their role in preventing HOA and THA.

Nowadays, THA is the sole therapeutic option for HOA and AVN. Indications for THA were fourth and fifth Dale scale grades and included severe persistent pain and inability to walk (5). It is still challenging because of the patient's young age, systemic disease, medications, multiple affected joints, bone loss, continuous growth, risk of perioperative infections, and absence of data regarding implant use and revision terms (25). The most prevalent diagnosis for THA in adulthood is primary osteoarthritis (66.1%), dysplastic hip (9.8%), osteonecrosis (9.8%), post-Perthes changes (2.5%), rheumatoid arthritis (1.9%), and other reasons, including outcomes of JIA (0.2%) (26). Fortunately, THA frequency in JIA patients, especially during childhood, decreased and shifted to an elderly group due to early diagnosis, biologic therapy, and corticosteroid avoidance (9, 27). Despite the expanding access to biologic therapy, unfortunately, many patients continue to receive corticosteroids. Long-term corticosteroid treatment, together with chronic inflammation, may lead to pathological bone and joint changes that mainly occur before growth is complete (5). Even at low doses, long-term corticosteroid therapy is associated with AVN risk in the general population, without nosology correlation (22). It was highlighted that 10 mg prednisolone daily was associated with a 6.7% increase in AVN rate. The cumulative boundary dose is 2,000 mg, and the high-risk dose is more than 10,000 mg (23). In our study, it was 2,700 mg-an independent risk factor of HOA development. The high risk of AVN development varies from 3 months to 1 year after corticosteroid therapy (10). It is interesting to note that if THA in JIA has occurred, the survival of the prosthesis is significantly poorer in patients receiving corticosteroids than those who had methotrexate (28). There are practically no recent investigations about corticosteroids and HOA correlation in JIA patients with biologic treatment.

The main limitations of the study are related to its retrospective nature. Authors could not influence steroid administration, dosage, and duration, as well as time to biologic administration. The extended study period included two subgroup patients: (i) ~before 2011 (no access to biologics and corticosteroid prevalence) and (ii) since 2011 (better access to biologics and fewer corticosteroids usage). A relatively high proportion of patients with THA in childhood might be considered a tail-effect of the first subgroup and access to THA. The absence of an extended follow-up observation period blinds the actual scope of the problem in adults with JIA. More patients with JIA will develop hip osteoarthritis and will have THA in the future, and the whole population with aging. In our study, the diagnosis of HOA was established according to Dale's radiographic JIA classification, but it was initially created for the knee and was not validated for the hip joint, so some misinterpretations were possible. The lowest age of the study inclusion in sJIA might underestimate the actual magnitude of the problem.

## Conclusion

Systemic JIA is a significant factor associated with HOA along with systemic corticosteroids and impaired calcium-phosphorus metabolism, as well as persistent inflammation, non-achievement of remission, and delay in biologic treatment initiation. The delayed hip involvement in systemic JIA allows considering HOA as a severe adverse event of corticosteroid therapy and a complication of sJIA itself. Strict monitoring of the systemic corticosteroids administration and disease control in JIA in general and in the systemic JIA, in particular, is required. It is necessary to avoid systemic corticosteroids in patients with non-systemic JIA and switch to biologic therapy as soon as possible, especially in corticosteroid-dependent or refractory patients. Corticosteroid administration should be limited to life-threatening conditions and must be tapered rapidly with early biologic intervention in sJIA. It is recommended to consider calcium-phosphorus metabolism, check vitamin D plasma level and prescribe therapeutic doses of vitamin D in patients treated with corticosteroids. Further investigation is required to clarify the mechanisms of HOA in JIA patients.

## Data Availability Statement

The original contributions presented in the study are included in the article/supplementary material, further inquiries can be directed to the corresponding author/s.

## Ethics Statement

The studies involving human participants were reviewed and approved by Ethical Committee of Saint Petersburg State Pediatric Medical University (protocol number 11/10 from 23.11.2020). Written informed consent to participate in this study was provided by the participants' legal guardian/next of kin.

## Author Contributions

LS and MK contributed to the conception and design of the study. LS, IA, RR, NL, SK, and MK organized the database and contributed equally to all of the following aspects of the manuscript: conception, acquisition of data, drafting, and revising the article. LS and MK performed the statistical analysis and wrote the first draft of the manuscript. IA, RR, NL, and SK wrote sections of the manuscript. All authors contributed to manuscript revision, read, and approved the submitted version.

## Funding

This work was financially supported by the Ministry of Science and Higher Education of the Russian Federation (Agreement No. 075-15-2020-901).

## Conflict of Interest

The authors declare that the research was conducted in the absence of any commercial or financial relationships that could be construed as a potential conflict of interest.

## Publisher's Note

All claims expressed in this article are solely those of the authors and do not necessarily represent those of their affiliated organizations, or those of the publisher, the editors and the reviewers. Any product that may be evaluated in this article, or claim that may be made by its manufacturer, is not guaranteed or endorsed by the publisher.
